# The Time to Most Recent Common Ancestor Does Not (Usually) Approximate the Date of Divergence

**DOI:** 10.1371/journal.pone.0128407

**Published:** 2015-08-14

**Authors:** James B. Pettengill

**Affiliations:** Division of rPublic Health Informatics and Analytics, Center for Food Safety and Applied Nutrition, US Food and Drug Administration, College Park, Maryland, United States of America; Field Museum of Natural History, UNITED STATES

## Abstract

With the advent of more sophisticated models and increase in computational power, an ever-growing amount of information can be extracted from DNA sequence data. In particular, recent advances have allowed researchers to estimate the date of historical events for a group of interest including time to most recent common ancestor (TMRCA), dates of specific nodes in a phylogeny, and the date of divergence or speciation date. Here I use coalescent simulations and re-analyze an empirical dataset to illustrate the importance of taxon sampling, in particular, on correctly estimating such dates. I show that TMRCA of representatives of a single taxon is often not the same as divergence date due to issues such as incomplete lineage sorting. Of critical importance is when estimating divergence or speciation dates a representative from a different taxonomic lineage must be included in the analysis. Without considering these issues, studies may incorrectly estimate the times at which historical events occurred, which has profound impacts within both research and applied (e.g., those related to public health) settings.

## Introduction

The development of novel analytical methods have enabled evolutionary biologists to extract a great deal of information from genetic data, including estimates of the date of divergence between sister taxa and the time to most recent common ancestor of a group of individuals [1, 2]. The utility of this information extends to applied research, where temporal information about, for example, hospital infections or the emergence of bacterial strains allows practitioners to better understand the evolution of pathogens [3, 4]. Such information in turn may result in more efficient control measures and accurate traceback investigations (i.e., linking clinical isolates to an environmental source).

A paper published in *PLoS Genetics* by Zhou et al. [5], “Neutral Genomic Microevolution of a Recently Emerged Pathogen, *Salmonella enterica* Serovar Agona”, describes the genetics and evolutionary history of a taxonomic group based on the analysis of whole genome sequence data. The authors conclude from analyses conducted with the program BEAST (Bayesian Evolutionary Analysis Sampling Trees) [2] that estimated the time to most recent common ancestor (TMRCA), which is the time at which alleles have coalesced to a single ancestor, that “Agona evolved in 1932” and “Agona is a recently evolved pathogen, which likely arose about 80 years ago. Consistent with this interpretation, Agona was first identified in 1952.” As these statements and the title of the article allude to, the authors infer from this that Agona ‘emerged’ or diverged around a century ago from a separately evolving independent lineage. However, the author’s incorrectly equated node age with TMRCA and more importantly the taxon sampling was insufficient to warrant conclusions about the date of emergence. The reason being that the study does not include a separate serovar (ideally the closest known serovar). As a result, the authors incorrectly equated TMRCA among the samples they analyzed to the date of divergence or ‘speciation’ date.

## Results and Discussion

### Coalescent simulations

To illustrate the evolutionary scenarios under which the TMRCA will not equal the date of divergence and instances where it might, I ran coalescent simulations with the ms program [6]. All ms simulations consisted of two populations, one of size 10 and the other a single individual, where at some point in the past they diverged (or looking backwards in time they coalesce to a common ancestor). This scenario reflects the general sampling design within many evolutionary and phylogenetic studies where there is dense sampling within a group of interest and a single representative from an outgroup is chosen to provide polarity to the tree. The only parameter that varied between scenarios was the time at which coalescence occurred. Under the first scenario, deep divergence, divergence occurred well into the past at 40 *N*_*0*_ generations (ms command: ms 11 1-t 2.0-I 2 1 10-ej 10 1 2 –T). Under the second scenario, recent divergence, divergence occurred relatively recently at only 0.04 *N*_*0*_ generations in the past (ms command: ms 11 1-t 2.0-I 2 1 10-ej 0.01 1 2 –T).

**Fig 1:**
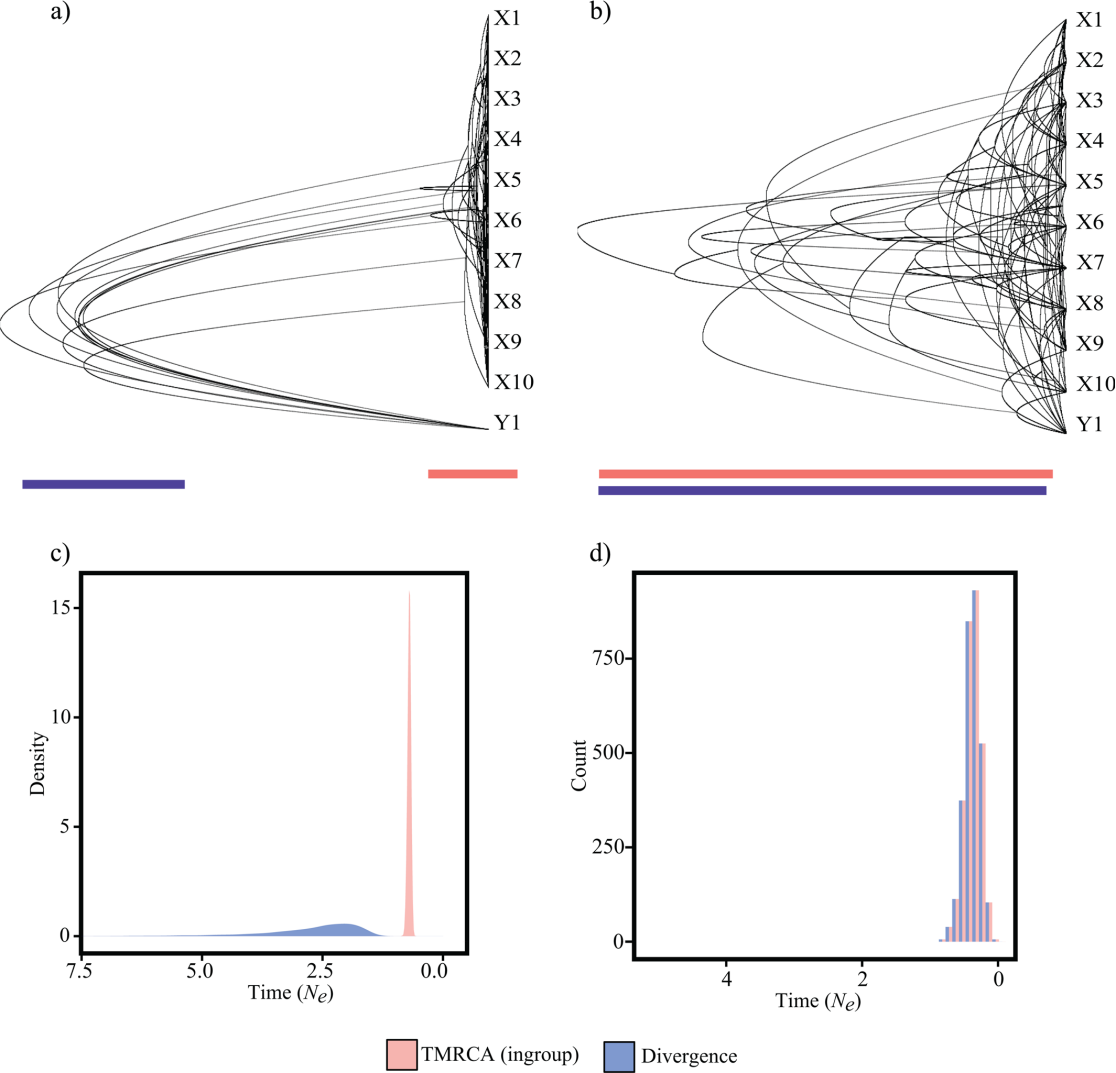
Ten randomly selected genealogies from the coalescent simulations of two taxa X (N = 10) and Y (N = 1) using the ms [6] program under the a) early divergence scenario where the TMRCA is much more recent than divergence, b) the late divergence scenario where TMRCA and divergence data overlap. Visualization was performed using DensiTree v2.2.1 [14]. The red line and blue line approximate the range of estimates for divergence time and TMRCA, respectively. Histograms of the distribution of estimates of the date of divergence between X and Y and TMRCA of X run under the c) early divergence and d) late divergence scenarios. In panel (d) bars are offset (“dodge”) to illustrate that the distributions for each parameter are indistinguishable.

Based on previous research, complete lineage sorting is expected to occur 4–7 *N*_*e*_ (the effective population size) generations after reproductive isolation [7] and, thus, under the deep divergence scenario, there is a clear split between the two groups due to sufficient time for the sorting of ancestral polymorphisms. This in turn shows that the TMRCA of the larger population is much more recent than the actual divergence time (Fig 1A and 1C). Thus, simulations under the early divergence model clearly illustrate the problem of equating the TMRCA among a set of individuals from the same taxon as the date of emergence. Such an assumption may greatly underestimate the date of divergence, which can only be inferred by including a separate independently evolving lineage (e.g., species or serovar). Below I also illustrate that this deep divergence scenario best represents the case of Agona from its sister taxa and, thus, equating TMRCA among a set of only Agona samples with divergence time in Zhou et al. was incorrect.

In contrast, under the recent divergence scenario, there is little consistency in the topologies where within many trees the larger ‘ingroup’ is not monophyletic and the TMRCA is indistinguishable from the divergence time (Fig 1B and 1D). Although under such a scenario the TMRCA may be quite similar to the divergence date, the possibility also exists that the TMRCA may predate the divergence time due to incomplete lineage sorting (e.g., [8]).

### Reanalysis of the Zhou et al. dataset

For simplicity, I ran BEAST analyses including only four samples (Table 1) from the original publication of Zhou et al. [5], which were arbitrarily chosen to capture the evolutionary breadth contained in that study. I also ran BEAST including the closest known serovar to Agona, *S*. Soerenga, which was identified based on a large phylogeny including 76 *S*. *enterica* ssp. *enterica* serovars [9]. Given that the SNP matrix within Zhou et al. [5] was not available, I downloaded the assemblies and performed a whole genome sequence alignment using Mugsy v.1.2.3 with default settings [10]. The program ClonalFrameML v1.25 [11] was then used to detect recombination, which can bias estimates of TMRCA and other evolutionary dates [1]. Multiple runs of ClonalFrameML were carried out varying the priors (e.g., R/Θ) to ensure stability of parameter estimates. Positions in the whole genome alignment file that contained ambiguous bases or missing data were excluded from the analysis via the ignore_incomplete_sites option in ClonalFrameML. A custom script was written to remove positions within the whole genome alignment file that were attributed to recombinational events in the analysis; this file was then culled to only include variant positions and was the SNP matrix used for analyses in BEAST v1.8.1. All runs with BEAST were of a sufficient number of generations to ensure adequate convergence (i.e., ESS > 200). See S1 for SNP matrices.

**Table 1:** Metadata for the five samples of Salmonella enterica ssp. enterica Serovar included in the empirical analyses.

Serovar	Tree ID	Strain	Year	Country	Reads	Project	Assemblies	Source
Agona	CARC 1952	WS0243	1952	Ghana	ERS180381	PRJEB1134	CARC01000001-203	Zhou et al. (2013)
Agona	CARK 2000	DBS_20001356	2000	Scotland	ERS180373	PRJEB1126	CARK01000001-169	Zhou et al. (2013)
Agona	CART 2008	MC_08–0610	2008	Ireland	ERS180363	PRJEB1116	CART01000001-146	Zhou et al. (2013)
Agona	CATS 2009	MC_09–0426	2009	Ireland	ERS180376	PRJEB1129	CATS01000001-167	Zhou et al. (2013)
Soerenga	S. Soerenga 2003	695	2003	USA	SRR652950	PRJNA78407	ASM48656v1	Timme et al. (2013)

**Fig 2:**
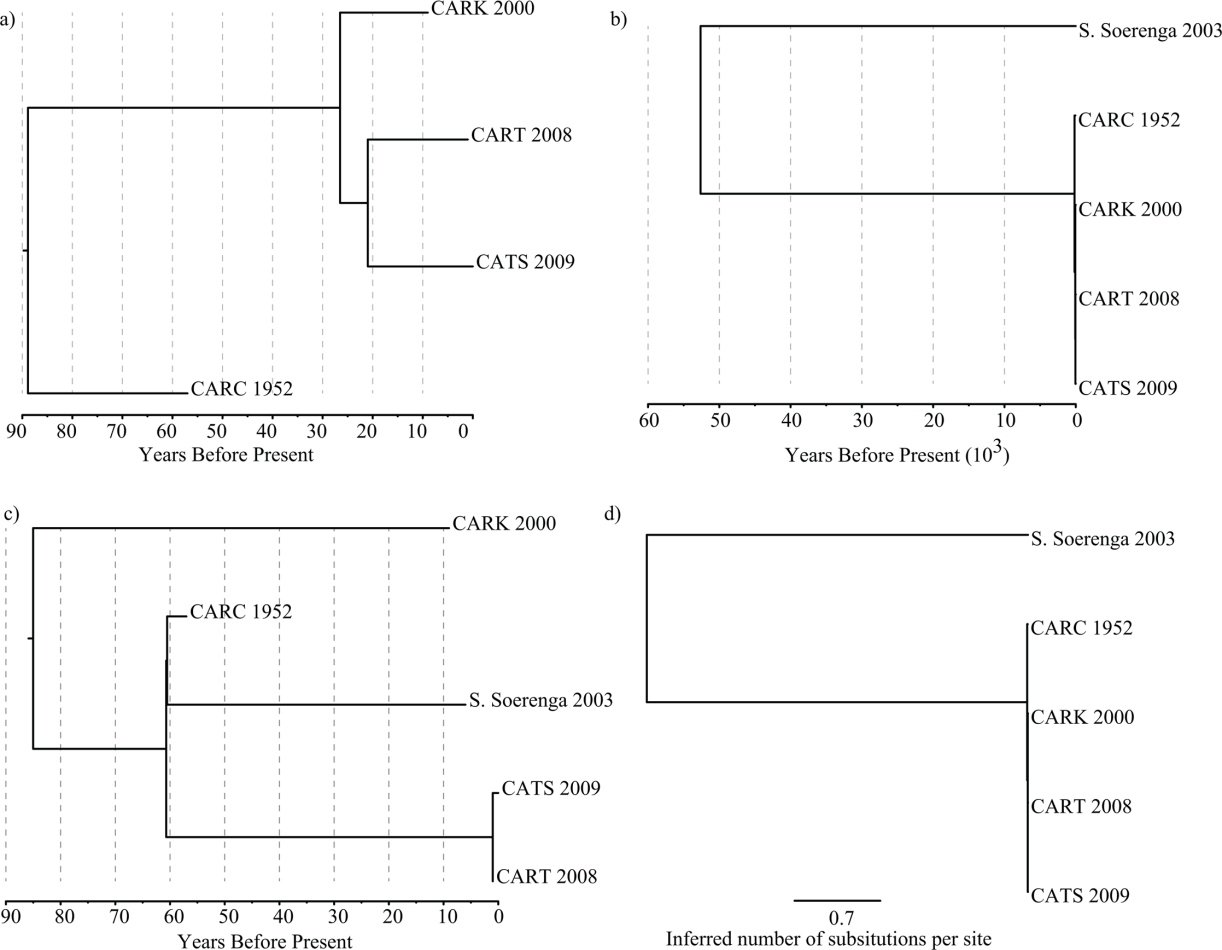
Genealogies under the different BEAST analyses where a) only Agona samples were included and analyzed with the best fitting model identified in Zhou et al. [5], b) with the outgroup analyzed with a strict molecular clock (note that the scale is in 10^3^ ybp) and, c) with the outgroup Soerenga and analyzed under best fitting model from the publication and, d) a phylogeny inferred with MrBayes [12].

I analyzed the four Agona samples contained in Zhou et al. [5] under the best fitting model described in the paper (e.g., uncorrelated lognormal clock rate and Gaussian Markov random fields (GMRF) tree model that allows for historical fluctuations in population size). Under this analysis, the age of the most basal node of the Agona isolates sampled was 1927 or 88 ybp (years before present) (CI95% 57–512 ybp) (Fig 2A), which is quite similar on an evolutionary scale to the year 1932 that was observed in Zhou et al. [5]. However, the mean estimate of the actual TMRCA (treeModel.rootHeight from the BEAST output) was nearly three times as old (313 ybp; 95%CI: 57–295 ybp) and illustrates the difference that exists between an estimate of the age of the most basal node in a phylogeny and an estimate of the time at which alleles segregating in the dataset coalesce back to a single common ancestor (i.e., the TMRCA). Had Zhou et al. [5] correctly identified the TMRCA their incorrect estimate of the date of emergence of Agona would have likely been hundreds of years older than what they reported, which was based on the age of the most basal node.

With the outgroup Soerenga included approximately 25% of the positions were attributed to recombination and once removed the SNP matrix contained 30359 SNP positions. Given the extremely high percentage of positions attributed to recombination when Soerenga was included I also performed the analyses on the original SNP matrix not accounting for recombination. With these matrices I ran two separate analyses: 1) a strict clock and GMRF tree model and 2) a relaxed lognormal clock and GMRF tree model like that used in Zhou et al. [5]. I also performed an analysis using MrBayes v3.2.1 [12] to provide a topology and branch lengths under an alternative method for inferring phylogenies; MrBayes was run with default settings and was monitored to ensure adequate convergence (i.e., the standard deviation of the split frequencies was well below 0.001).

Under the strict clock and using the SNP matrix with recombination removed, it is clear that the TMRCA does not equal the divergence time. The TMRCA was 521 ybp (95%CI: 74–940) and the divergence date was approximately 50,000 ybp (95%CI: 16,000–340,980 ybp) years older than the former (Fig 2B). When recombination was not accounted for the TMRCA was 690 ybp (95%CI: 233–4297 ybp) and divergence between Agona and Soerenga occurred 30,000 ybp (95%CI: 10087–194740). Not surprisingly the height of the node uniting all Agona samples that Zhou et al. equated with TMRCA is also older (168 ybp) than when Soerenga was not included (115 ybp).

Under the relaxed lognormal and GMRF model, the results are difficult to interpret as Agona is no longer monophyletic (Fig 2C); this was also the case when analyzing the SNP matrix that did not account for recombination. It is important to note that the polyphyly of Agona in those analyses is not an artifact of inappropriate rooting as the root is automatically inferred within a BEAST analysis. As a result, the relaxed clock model appears to be problematic under the evolutionary history of Agona and Soerenga. This is most likely due to under such a model there are widely different rates being applied in an attempt to account for the large evolutionary distance between the two groups. This may also explain the very small timeframe of divergence under such a model (Fig 2C). Although the rates of evolution within the dataset may not be clock-like, a standard deviation for clock rate of 3.96 in log space is highly unlikely to be biologically true as is the polyphyly of Agona with respect to Soerenga.

## Conclusions

From the results presented here, it is clear that the emergence of Agona was not within the past century if for no other reason than in Zhou et al. they interpreted the age of the most basal node in a phylogeny as the TMRCA, which is not correct; the former will always be younger than the latter. Based on the re-analysis of the Zhou et al. data including the closest known sister taxon to Agona the date of divergence may be as old as tens of thousands of years before present. This estimate likely represents an upper bound on the date of divergence for two reasons. First, with additional taxon sampling a different serovar may be found to be sister to Agona, which would bring the estimate of divergence or speciation of Agona closer to the present. Second, the estimate of emergence of Agona obtained here may be an overestimate due to time dependency of rate variation and the potential biases introduced by inferring more distant evolutionary dates based on heterochronous sampling [13]. However, despite these limitations, one cannot rest on taxon sampling of a single taxonomic group to estimate dates of emergence but must include members of a separate independently evolving lineage (e.g., sister serovar or species). Furthermore, node age does not equal TMRCA. In conclusion, the TMRCA will often not be similar to divergence time and by assuming they are one risks making erroneous conclusions about patterns of evolutionary history that also may have negative consequences for public health (e.g., dating transmissions events within a disease outbreak).

## Supporting Information

S1 File A zipped archive of the SNP matrix with recombination removed that was analyzed with BEAST and MrBayes. (ZIP)
